# Apelin and Copeptin Levels in Patients With Chronic SIAD Treated With Empagliflozin

**DOI:** 10.1210/jendso/bvae106

**Published:** 2024-05-29

**Authors:** Sophie Monnerat, Nikolaos Drivakos, Fiona A Chapman, Neeraj Dhaun, Julie Refardt, Mirjam Christ-Crain

**Affiliations:** Department of Endocrinology, Diabetology and Metabolism, University Hospital Basel, 4031 Basel, Switzerland; Department of Endocrinology, Diabetology and Metabolism, University Hospital Basel, 4031 Basel, Switzerland; Department of Clinical Research, University of Basel, 4031 Basel, Switzerland; Department of Nephrology, Hospital Center of Biel, 2501 Biel, Switzerland; BHF/University of Edinburgh Centre for Cardiovascular Science, Queen's Medical Research Institute, Edinburgh EH16 4TJ, UK; Department of Renal Medicine, Royal Infirmary of Edinburgh, Edinburgh EH16 4SA, UK; BHF/University of Edinburgh Centre for Cardiovascular Science, Queen's Medical Research Institute, Edinburgh EH16 4TJ, UK; Department of Renal Medicine, Royal Infirmary of Edinburgh, Edinburgh EH16 4SA, UK; Department of Endocrinology, Diabetology and Metabolism, University Hospital Basel, 4031 Basel, Switzerland; Department of Endocrinology, Diabetology and Metabolism, University Hospital Basel, 4031 Basel, Switzerland; Department of Clinical Research, University of Basel, 4031 Basel, Switzerland

**Keywords:** apelin, copeptin, empagliflozin, hyponatremia, SIAD

## Abstract

**Background:**

Empagliflozin increases sodium levels in patients with a chronic syndrome of inappropriate antidiuresis (SIAD), and dapagliflozin increases apelin levels in patients with diabetes mellitus. Exogenous apelin increases sodium levels in rats with SIAD. We aimed to investigate whether an increase in plasma apelin concentration may contribute to the efficacy of empagliflozin in SIAD.

**Methods:**

Post hoc secondary analysis of a double-blind, crossover, placebo-controlled trial performed from December 2017 to August 2021 at the University Hospital Basel, Switzerland, investigating the effect of 4-week treatment with empagliflozin 25 mg/day as compared to placebo in 14 outpatients with chronic SIAD (NCT03202667). The objective was to investigate the effect of empagliflozin on plasma apelin and copeptin concentrations and their ratio.

**Results:**

Fourteen patients, 50% female, with a median [interquartile range] age of 72 years [65–77] were analyzed. Median apelin concentration was 956 pmol/L [853, 1038] at baseline. Median [interquartile range] apelin relative changes were +11% [0.7, 21] and +8% [−5, 25] (*P* = .672) at the end of the placebo and empagliflozin phases, respectively. Median copeptin concentration was 2.6 [2.2, 4.5] pmol/L at baseline and had a relative change of +5 [−2. 11]% and +25% [10, 28] (*P* = .047) over the placebo and empagliflozin phases, respectively.

**Conclusion:**

Empagliflozin did not lead to significant changes in apelin or the apelin/copeptin ratio in patients with chronic SIAD but led to an increase in copeptin. This suggests that the efficacy of empagliflozin in SIAD is independent of apelin and is not blunted by the adaptative increase in copeptin.

Hyponatremia is the most common electrolyte disorder encountered in clinical practice [1] and is associated with increased morbidity and mortality [2]. The syndrome of inappropriate antidiuresis is one of the most common causes of hyponatremia [3]. It is characterized by hypotonic euvolemic hyponatremia and hemodynamically and osmotically inappropriate increased arginine vasopressin (AVP) activity. This results in the inability to produce dilute urine and in free water retention [3].

Apelin is an endogenous peptide hormone produced in several organs and tissues, such as the brain, the heart, the vascular endothelium, and the kidneys [4-6], and is involved in cardiovascular regulation and energy metabolism as well as water homeostasis [7-10]. The latter is regulated by apelin and AVP in opposing ways: apelin promotes diuresis and vasodilatation whereas AVP promotes antidiuresis and vasoconstriction [11]. This functional crosstalk is mirrored by the colocalization of both hormones and their respective receptors. AVP and apelin are secreted from neurosecretory cells of the hypothalamic supraoptic and paraventricular nuclei and apelin receptors [12] and arginine vasopressin receptors (AVPR)1a/1b are expressed by magnocellular AVP neurons [13]. In the kidneys, apelin receptors are found in the glomeruli and in all nephron segments including the collecting duct where AVPR2 are also expressed [14, 15]. In states of water depletion and hyperosmolality, AVP is released by the posterior pituitary. It then binds to AVPR2 on principal cells in the renal collecting duct, activating the cAMP/protein kinase A pathway and leading to insertion of aquaporin 2 in the apical membrane and free water retention [16]. In a state of hypoosmolality, apelin acts in opposition by inhibiting the secretion of AVP from the posterior pituitary gland [17], increasing renal blood flow, and negating the effect of AVP on the kidneys [18]. In lactating rats, intravenous apelin administration reduces aquaporin 2 insertion in the apical membrane of the collecting duct cells by inhibiting the intracellular production of cAMP and calcium influx, thus increasing diuresis and reducing urine osmolality [19].

Patients with syndrome of inappropriate antidiuresis (SIAD) have an altered balance between apelin and copeptin with disproportionately increased levels of copeptin, an equimolar surrogate marker of AVP [20], while levels of apelin are only slightly increased [21]. An abnormal apelin/vasopressin balance may thus contribute to water retention in patients with SIAD [21]. In support of this, a study in rats with SIAD showed that reestablishing a physiological apelin/copeptin ratio with the administration of exogenous apelin increases urinary output and plasma sodium concentration [22]. Whether this approach is also effective in patients with SIAD is currently being investigated in a double-blind placebo-controlled crossover study (NCT06277336).

We demonstrated in 2 randomized double-blind placebo-controlled trials that the sodium-glucose cotransporter 2 (SGLT2) inhibitor empagliflozin is a promising treatment option for patients with SIAD [23, 24]. One month of treatment with empagliflozin led to a sodium increase of 4 mmol/L in comparison to placebo [24]. Others have shown that the SGLT2 inhibitor dapagliflozin increased apelin levels by up to 18% in patients with type 2 diabetes mellitus and heart failure [25]. To our knowledge, the effect of SGLT2 inhibitors on the concentration of apelin and apelin/copeptin ratio in patients with SIAD is unknown. We hypothesized that SGLT2 inhibitors increase apelin levels in SIAD. To investigate this, we performed a secondary analysis of our crossover trial in outpatients with chronic SIAD [24] and aimed to characterize the course of apelin levels and of the apelin/copeptin ratio during 4 weeks of treatment with empagliflozin.

## Materials and Methods

### Study Design and Participants

This is a post hoc secondary analysis of a prospective randomized, crossover, double-blind, placebo-controlled trial performed at the University Hospital Basel, Switzerland from December 2017 to August 2021. The competent ethics committee (EKNZ 2017-00701) and the national agency for the authorization and supervision of therapeutic products (Swissmedics 2017DR2127) approved the study protocol and study medication. The study was registered at ClinicalTrials.gov (NCT03202667) and complied with the principles of the Declaration of Helsinki.

The main eligibility criteria included being ≥18 years old, being diagnosed with chronic SIAD defined as plasma sodium concentration <135 mmol/L, euvolemia on clinical examination, plasma osmolality <275 mOsm/kg, urine osmolality >100 mOsm/kg, urine sodium >30 mmol/L, the exclusion of uncontrolled hypothyroidism and hypocortisolism. Exclusion criteria included severe symptomatic or transient hyponatremia, pregnancy or breastfeeding, estimated glomerular filtration rate < 45 mL/min/1.73 m^2^, type 1 diabetes mellitus, liver cirrhosis or acute hepatic impairment (aspartate amino transferase/alanine amino transferase > 3x upper limit of normal), heart failure, or being treated with SGLT2 inhibitors, lithium chloride, urea, or glitazone.

The investigational medical products consisted of encapsulated empagliflozin 25 mg and matched to a visually corresponding placebo. Patients were randomly allocated to 28 days treatment with empagliflozin followed by 28 days treatment with placebo or vice versa. Further treatment included limitation of daily fluid intake to ≤1.5 L/day. Further details can be found in the initial report of the study [24].

### Laboratory Markers

Fasting blood samples were drawn between 8:00 and 11:00 Am, and fresh serum and plasma aliquots were stored at −80 °C until batch analysis. Apelin was measured using a commercial enzyme-linked immunosorbent assay kit (Phoenix Pharmaceuticals Cat# EK-057-23, RRID:AB_3096967) with a lower limit of detection 0.07 ng/mL and intra- and interassay variation of <10% and <15%, respectively. Copeptin was measured with the CE certified automated immunoassay BRAHMS Copeptin proAVP assay (Thermo Fisher Scientific B.R.A.H.M.S Cat# 857050N, RRID:AB_3073917) with a coefficient of variation within-laboratory precision of approximately 9.8% and a coefficient of variation for reproducibility of 7%. Baseline values correspond to the first visit of the first treatment phase. The apelin/copeptin ratio was calculated by dividing the apelin by the copeptin concentration.

### Study Objectives and Outcomes

The primary objective was to investigate the effect of empagliflozin on apelin concentration. Secondary objectives were to investigate the effect of empagliflozin on copeptin levels and on the apelin/copeptin ratio as well as to identify predictors for changes in apelin.

The primary outcome was the absolute change in plasma apelin concentration (pmol/L) at the end of the empagliflozin phase compared to the end of the placebo phase. Secondary outcomes were the relative and absolute changes in apelin concentration, copeptin concentrations, and apelin/copeptin ratio after 1 and 4 weeks of treatment; the relationship between apelin and sodium concentrations; and the adjusted relationship between empagliflozin and the changes at week 4 in apelin concentrations, copeptin concentrations, and their ratio.

### Statistical Analysis

Baseline characteristics are summarized using descriptive statistics. Discrete variables are expressed as frequencies [percentage (%) and number of patients (n)]. Continuous variables are expressed as median and interquartile range (IQR). Changes in apelin and copeptin are represented graphically with boxplots.

Absolute and relative changes in apelin, copeptin, and their ratio were compared using a paired Wilcoxon signed-rank test. The relationships between apelin, apelin/copeptin ratio, and sodium concentrations were investigated by computing Spearman correlation coefficients. The association between changes in apelin, copeptin, and their ratio and empagliflozin was further investigated by fitting a linear mixed-effects model [26, 27] with changes in the laboratory parameters as the outcome variable, patients as the random effect, and the following fixed effects: the respective baseline value, age, sex, and treatment with an angiotensin converting enzyme (ACE) inhibitor or angiotensin II receptor blocker (ARB). As there was no evidence for either carryover or sequence effect, treatment sequence and study phase were not included in the final models. A linear mixed regression model was used to analyze changes in sodium levels, for which details are found in the main report of this study [24].

All analyses were performed using the statistical program R (version 4.2.3 [28]). A two-sided significance level of 0.05 was used for every analysis and there was no correction for multiple testing.

## Results

### Baseline Characteristics

Seventeen patients were included in the initial study, of whom 14 completed both treatment cycles and form the intention-to-treat analysis set used in the main report of the study [24] and this analysis. Seven (50%) were female, and median age [IQR] was 72 [65–77] years. SIAD was caused by diseases of the central nervous system (n = 2, 14%), medications (n = 4, 29%), pulmonary diseases (n = 3, 21%), and chronic pain (n = 1, 7%) and was idiopathic in 4 patients (29%). Patients had been hyponatremic for a median [IQR] of 46 months [16–57] and median [IQR] plasma sodium concentration at inclusion was 131 mmol/L [130, 132] (Table 1). Nine patients (64%) took antihypertensive medication, ie, 1 (7%) took ACE inhibitors, 7 (50%) took ARB, 5 (36%) took beta blockers, 5 (36%) took calcium channel antagonists, and 2 (14%) took thiazide diuretics. Detailed baseline characteristics are shown in Table 1.

**Table 1.** Baseline characteristics

Characteristics	Participants n = 14
Demographics	
Age, years	72 [65, 77]
Ethnicity: Caucasian, n (%)	14 (100)
Sex: female, n (%)	7 (50)
Comorbidities	
Active smoking, n (%)	3 (21)
Alcohol consumption, n (%)	11 (79)
Arterial hypertension, n (x)	11 (79)
Asthma, n (%)	2 (14)
Chronic heart failure, n (%)	0 (0)
COPD, n (%)	1 (7)
Depression, n (%)	2 (14)
Hepatic insufficiency, n (%)	0 (0)
Osteoporosis, n (%)	2 (14)
Renal insufficiency, n (%)	0 (0)
Subarachnoid hemorrhage, n (%)	1 (7)
Tuberculosis, n (%)	1 (7)
Type 2 diabetes mellitus, n (%)	2 (14)
SIAD causes n (%)	
Central nervous system disorders	2 (14)
Drug-induced	4 (29)
Idiopathic	4 (29)
Pulmonary disease	3 (21)
Stress (chronic pain)	1 (7)
Duration of hyponatremia, months	46 [16–57]
Medications	
Antidepressants, n (%)	3 (21)
Antihypertensive, n (%)	9 (64)
ACE inhibitor	1 (7)
ARB	7 (50)
Beta blocker	5 (36)
Calcium channel antagonist	5 (36)
Diuretics	2 (14)
NSAID, n (%)	1 (7)
Opioid agonist, n (%)	1 (7)
Sodium chloride tablets, n (%)	1 (7)
Vital signs	
BMI, kg/m^2^	24.5 [21.6, 27.6]
Blood pressure, mmHg	
Diastolic	80 [69, 86]
Systolic	149 [135, 160]
Heart rate, bpm	66 [61, 72]
Height, cm	170 [164, 176]
Volume status, euvolemia	14 (100)
Weight, kg	72 [69, 80]
Laboratory parameters	
eGFR, mL/min/1.73 m^2^	87 [85, 101]
Plasma creatinine, µmol/L	61 [58, 65]
Plasma glucose, mmol/L	5 [4.8, 5.8]
Plasma osmolality, mOsm/kg	272 [264, 281]
Plasma sodium, mmol/L	131 [130, 132]
Serum cortisol, nmol/L	369 [291, 429]
Serum TSH, mlU/L	1.5 [1.05, 2.96]
Urine osmolality, mOsm/kg	468 [361, 567]
Urine sodium, mmol/L	98 [77, 130]

Categorical variables are shown as frequencies (%), numerical variables as median [interquartile range].

Abbreviations: ACE, angiotensin-converting enzyme; ARB, angiotensin II receptor blocker; BMI, body mass index; bpm, beat per minute; COPD, chronic obstructive pulmonary disease; eGFR, estimated glomerular filtration rate; NSAID, nonsteroidal anti-inflammatory drugs; SIAD, syndrome of inappropriate antidiuresis.

### Apelin

**Figure 1.**
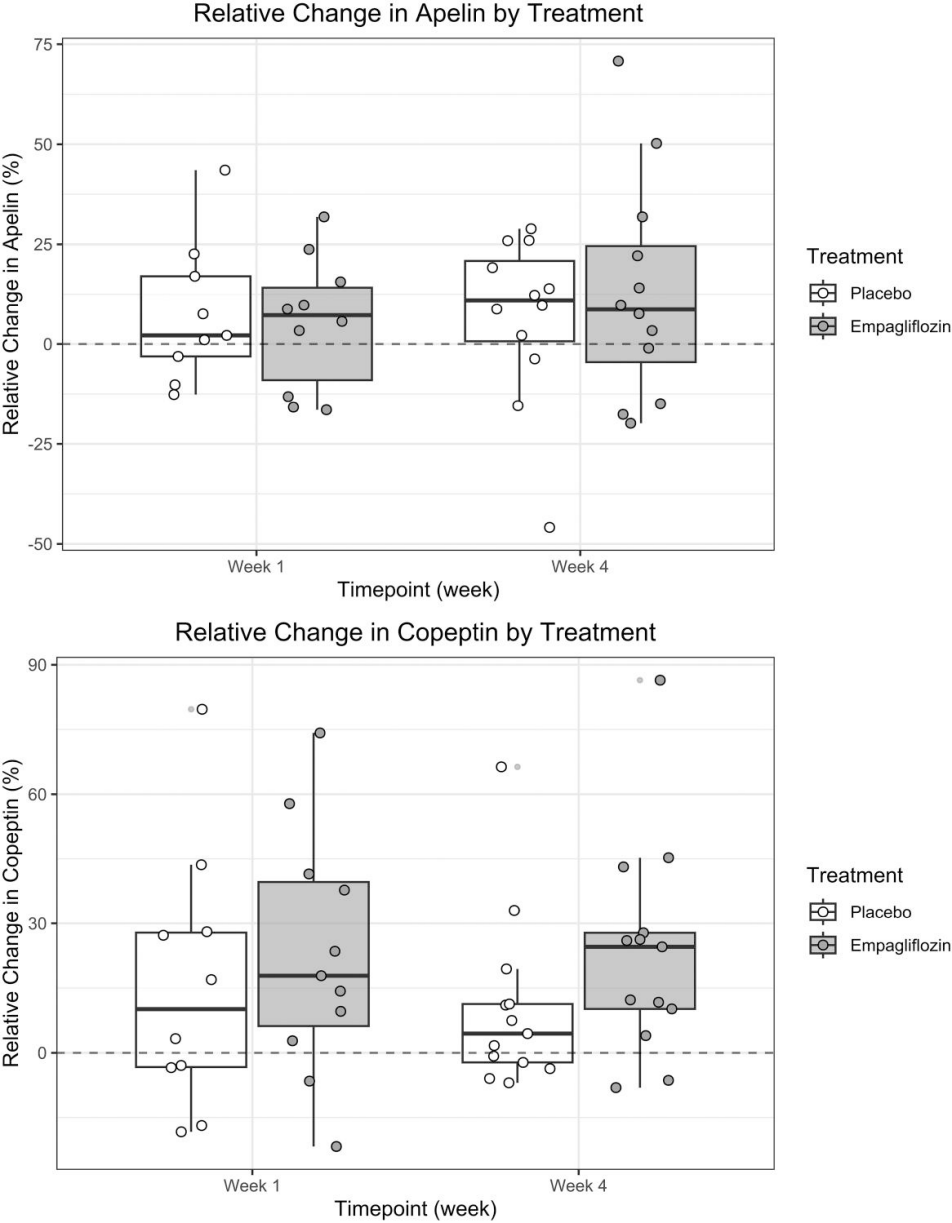
Changes in apelin and copeptin by treatment. Changes in laboratory parameters were computed by calculating the relative change from baseline to week 1 and 4 respectively. Values above the dashed line represent an increase and values below the dashed line a decrease from baseline.

Median [IQR] plasma apelin concentration was 956 [853, 1038] pmol/L at baseline (n = 13), 1080 [825, 1218] pmol/L at the end of the placebo phase (n = 14), and 1115 [848, 1275] pmol/L at the end of the empagliflozin phase (n = 13) (Table 2). This corresponds to an absolute change of +102 [1, 166] pmol/L and +82 [−47, 199] pmol/L (*P* = .677) and a relative change of +11% [0.7, 21] and +8% [−5, 25] (*P* = .672) during the placebo and empagliflozin phases, respectively (Fig. 1). Baseline apelin concentration did not correlate with baseline sodium concentration (ρ = 0.34, *P* = .282), nor at their absolute changes at week 4 (ρ = −0.007, *P* = .974; Supplementary Fig. S1) [29]. There was no significant association between changes in plasma apelin and age, sex, ACE inhibitor/ARB, or empagliflozin (Supplementary Table S2) [29], despite a tendency toward greater apelin increase upon empagliflozin in patients treated with ACE inhibitor or ARB (Supplementary Fig. S2) [29].

**Table 2.** Apelin and copeptin concentrations by timepoint and treatment

Treatment		Placebo		Empagliflozin	
Timepoint	Baseline	Week 1	Week 4	Week 1	Week 4
Plasma apelin (pmol/L)	956 [853, 1038]*^a^*	1076 [813, 1232]*^b^*	1080 [825, 1218]	1057 [783, 1239]*^b^*	1115 [848, 1275]*^a^*
Serum copeptin (pmol/L)	2.6 [2.2, 4.5]*^a^*	3.7 [2.7, 6.4]*^b^*	3.2 [2.3, 6.1]	3.6 [2.6, 6.4]*^c^*	3.8 [2.9, 5.7]
Apelin/copeptin ratio	369 [147, 503]*^a^*	232 [153, 412]*^b^*	328 [151, 462]	317 [124, 427]*^b^*	284 [155, 390]*^a^*

Median [interquartile range] concentration of plasma apelin concentrations, plasma copeptin, and their ratio at baseline and over the 2 treatment phases. The apelin/copeptin ratio was calculated by dividing apelin concentration by copeptin concentration for each patient.

^*a*^n = 13.

^*b*^n = 11.

^*c*^n = 12.

### Copeptin

Median [IQR] plasma copeptin concentration was 2.6 [2.2, 4.5] pmol/L at baseline (n = 13), 3.2 [2.3, 6.1] pmol/L at the end of the placebo phase (n = 14), and 3.8 [2.9, 5.7] pmol/L at the end of the empagliflozin phase (n = 14) (Table 2). There was no significant difference in the absolute change [placebo: +0.2 [−0.1, 0.6] pmol/L; empagliflozin: +0.6 [0.3, 0.9] pmol/L; (*P* = .069)] but a significant difference in the relative change in copeptin, ie, +5 [−2. 11]% in the placebo and +25% [10, 28] in the empagliflozin phase (*P* = .047) (Fig. 1). Baseline copeptin concentration did not correlate with baseline sodium concentration (ρ = 0.059, *P* = .849), nor did their absolute changes at week 4 (ρ = 0.088, *P* = .671; Supplementary Fig. S1) [29]. There was no significant association between changes in copeptin and age, sex, or ACE inhibitor/ARB, but there was a tendency toward a greater change in copeptin upon empagliflozin treatment (β = 0.396; 95% confidence interval: 0.027, 0.766; *P* = .058) (Supplementary Table S2) [29].

### Apelin/Copeptin Ratio

Median [IQR] apelin/copeptin ratio was 369 pmol/pmol [147, 503] at baseline (n = 13), 328 pmol/pmol [151, 462] at the end of the placebo phase (n = 14), and 284 pmol/pmol [155, 390] at the end of the empagliflozin phase (n = 13) (Table 2). This corresponds to an absolute change of +8 pmol/pmol [−97, 36] and −4 pmol/pmol [−72, 32] (*P* = .266) and a relative change of +5% [−24, 13] and −4% [−26, 12] (*P* = .569) during the placebo and empagliflozin phases, respectively. Baseline apelin/copeptin ratio did not correlate with baseline sodium concentration (ρ = −0.06, *P* = .853), nor did their absolute changes at week 4 (ρ = −0.14, *P* = .507; Supplementary Fig. S1) [29]. There was no significant association between changes in apelin/copeptin ratio and age, sex, ACE inhibitor/ARB, or empagliflozin (Supplementary Table S2) [29].

## Sodium

Median [IQR] serum sodium level at baseline was 131 mmol/L [130–132]. After a 28-day treatment with empagliflozin, median [IQR] serum sodium levels increased to 134 mmol/L [132–136], while no increase was seen after 28 days of placebo (130 mmol/L [128–132]). This corresponds to a group difference of 4.1 mmol/L (95% confidence interval: 1.7-6.5; *P* = .004) [24]. Additional details can be found in the primary report of this study [24].

## Discussion

Our analysis has 2 main findings. First, 4 weeks treatment with empagliflozin had no effect on plasma apelin concentration. Second, copeptin—the surrogate marker of AVP—increased by a median of 25% upon empagliflozin, but this increase did not correlate with changes in plasma sodium. To our knowledge, we provide the first cross-over data on the effect of empagliflozin on apelin, copeptin, and apelin/copeptin ratio in patients with chronic SIAD.

Apelin inhibits AVP pituitary secretion and AVP-mediated antidiuresis and thus acts as its antagonist in salt and water homeostasis [18]. A cross-sectional study by Blanchard et al showed a higher copeptin to apelin ratio despite higher apelin concentrations in patients with SIAD as compared to healthy controls [21], suggesting not only increased AVP activity but also insufficiently increased apelin contributes to antidiuresis in these patients. In their study, apelin levels were more than 3 times lower and apelin/copeptin ratio about 8 times lower than the baseline values of our cohort. This is probably due to the discrepancy of the assays used in the 2 studies [30]. Sex differences in apelin levels remain elusive. One study in healthy volunteers and patients with chronic kidney disease (CKD) found 60% higher plasma apelin concentrations in women than in men [15], and another study showed that female patients with osteoarthritis had higher apelin levels than men [31]. In contrast, we did not find a sex difference in SIAD patients, and Blanchard and colleagues also found comparable apelin levels in healthy men and women of more than 60 years of age (age group of our cohort). In comparison, apelin concentrations were higher in younger men than in younger women in their study [21].

The therapeutic potential of increasing apelin levels and thus restoring a physiological apelin to copeptin ratio is supported by a recent preclinical study, in which the exogenous subcutaneous administration of a long-acting apelin-17 analogue in rats with SIAD increased urinary output and plasma sodium concentrations [22]. A recent uncontrolled intervention study in 153 patients with type 2 diabetes mellitus and heart failure showed that 6 months of treatment with the SGLT2 inhibitor dapagliflozin led to an 18% increase in apelin levels [25]. Furthermore, colocalization of apelin, its receptor, and SLGT2 has been demonstrated in the human kidney [15]. We therefore hypothesized that a similar increase in apelin could occur in patients with SIAD treated with empagliflozin and could thus contribute to augment diuresis in a glucose-independent manner. Contrary to our hypothesis, we found no difference in apelin levels at the end of the 4-week treatment with empagliflozin as compared to the 4-week treatment with placebo (Fig. 1). These differing findings could originate from the use of different assays and the difference in treatment duration and the administered SGLT2 inhibitor. In addition, the increase in apelin in patients with heart failure might have been elicited by improved cardiac function [25]. Moreover, our hypothesis that patients with higher apelin concentrations would have higher sodium concentrations at baseline and that a greater increase in apelin during treatment would lead to a greater sodium increase was also not confirmed by our data.

SIAD is characterized by an increase in AVP activity, which either relies on increased entopic or ectopic AVP secretion or increased AVP renal sensitivity depending on its etiology [32, 33]. Measurement of the AVP-surrogate marker copeptin is the gold standard to diagnose AVP-deficiency [34, 35] but has negligeable diagnostic value in hyponatremia because of an important overlap between etiologies, confounders increasing copeptin levels like stress and illness, and the high variability of copeptin levels in SIAD [36-38]. The latter is the reason why median baseline copeptin levels in our analysis (2.6 pmol/L) were much lower than in hospitalized patients with profound SIAD in 1 of our previous observational studies (11.9 pmol/L) [36]. The lower copeptin levels might also be due to the milder hyponatremia. However, we found no correlation between baseline copeptin and baseline sodium concentrations. Moreover, a third of the cohort had a drug-induced SIAD, in which drugs can directly stimulate AVPR2 and in which therefore, copeptin levels are expected to be low [33]. We observed an increase in copeptin at the end of the 4-week treatment with empagliflozin (Fig. 1), which we interpret as a hemodynamic AVP response secondary to glucose-induced osmotic diuresis. Importantly, we found no correlation between copeptin increase and sodium increase; therefore this increase in copeptin does not seem to blunt treatment efficacy. An increase in copeptin as an adaptive response to SGLT2 inhibitors is consistent with the findings of our first 4-day placebo-controlled trial in hospitalized patients with SIAD [23, 39] and previous studies in type 1 and type 2 diabetic patients under SGLT2-inhibitor treatment [40, 41].

The apelin system (apelin and its receptor) is currently being investigated as a possible treatment target for CKD and its associated cardiovascular diseases (ClinicalTrials.gov: NCT03956576) as it counteracts the renin-angiotensin-aldosterone system (RAAS), reduces blood pressure, and has an anti-inflammatory and antifibrotic effect [10, 42]. Whether an apelin agonist could offer additional benefits to current renoprotective treatments such as RAAS- and SGLT2-inhibitiors would be of great interest. We observed a tendency toward a greater apelin change with empagliflozin in SIAD patients already treated with RAAS inhibitors. One could hypothesize that combining RAAS and SGLT2 inhibition could offer additional synergistic benefits through an increase in apelin. Dual RAAS and SGLT2 inhibitor therapy has already demonstrated benefits in terms of disease progression and survival in patients with CKD and albuminuria [43]. It is known that treatment with SGLT2 inhibitors has a plethora of favorable effects (pleiotropic effects) [44]: cardiorenal protection, blood pressure and albuminuria reduction, lipid profile alteration, effects in myocardial metabolism [45], and even modulation of inflammatory response in acute myocardial infarction [46]. Further studies are needed to confirm the effect of a double inhibition of RAAS and SGLT2 on the apelin system and whether there is a resulting apelin increase associated with the positive effects of this combination therapy. Furthermore, clinical studies are needed to show whether a combination of RAAS inhibition, SGLT2 inhibition, and apelin agonism have an additive, clinically relevant effect in patients with cardiovascular disease and CKD.

Our analysis has several limitations. First, our sample size was modest and not powered for the current analysis. Second, the 4-week treatment might not have been long enough to lead to significant changes in apelin. Nevertheless, the main strength of our study relies in its cross-over and double-blind placebo-controlled design, providing a high internal validity in a difficult cohort of multimorbid and polymedicated patients.

## Conclusion

Treatment with empagliflozin in patients with chronic SIAD did not lead to significant changes in plasma apelin concentrations but led to an increase in copeptin, which did not blunt its efficacy in increasing sodium levels. Our findings do not support an empagliflozin-mediated increase in apelin to be involved in its efficacy in treating SIAD. Despite apelin not being influenced by SGLT2 inhibition in this setting, its therapeutic potential in treating SIAD remains intact and should be investigated in further research, including its changes following the use of other SIAD treatments, particularly vaptans, given the interplay between AVP and apelin. The ongoing double-blind placebo-controlled trial (ESCAPE Trial, ClinicalTrials.gov: NCT06277336) will provide additional insights into the effect of apelin administration in SIAD.

## Data Availability

Some or all datasets generated during and/or analyzed during the current study are not publicly available but are available from the corresponding author on reasonable request.

## Clinical Trial Information

Registration Number: NCT03202667.

## References

[1] Burst V . Etiology and epidemiology of hyponatremia. Front Horm Res. 2019;52:24‐35. 32097911 10.1159/000493234

[2] Peri A . Morbidity and mortality of hyponatremia. Front Horm Res. 2019;52:36‐48. 32097927 10.1159/000493235

[3] Esposito P, Piotti G, Bianzina S, Malul Y, Dal Canton A. The syndrome of inappropriate antidiuresis: pathophysiology, clinical management and new therapeutic options. Nephron Clin Pract. 2011;119(1):c62‐c73; discussion c73. 21677440 10.1159/000324653

[4] Medhurst AD, Jennings CA, Robbins MJ, et al Pharmacological and immunohistochemical characterization of the APJ receptor and its endogenous ligand apelin. J Neurochem. 2003;84(5):1162‐1172. 12603839 10.1046/j.1471-4159.2003.01587.x

[5] Katugampola SD, Maguire JJ, Matthewson SR, Davenport AP. [(125)I]-(Pyr(1))Apelin-13 is a novel radioligand for localizing the APJ orphan receptor in human and rat tissues with evidence for a vasoconstrictor role in man. Br J Pharmacol. 2001;132(6):1255‐1260. 11250876 10.1038/sj.bjp.0703939 PMC1572672

[6] Kleinz MJ, Skepper JN, Davenport AP. Immunocytochemical localisation of the apelin receptor, APJ, to human cardiomyocytes, vascular smooth muscle and endothelial cells. Regul Pept. 2005;126(3):233‐240. 15664671 10.1016/j.regpep.2004.10.019

[7] Murali S, Aradhyam GK. Structure-function relationship and physiological role of apelin and its G protein coupled receptor. Biophys Rev. 2023;15(1):127‐143. 36919024 10.1007/s12551-023-01044-x PMC9995629

[8] Wysocka MB, Pietraszek-Gremplewicz K, Nowak D. The role of apelin in cardiovascular diseases, obesity and cancer. Front Physiol. 2018;9:557. 29875677 10.3389/fphys.2018.00557 PMC5974534

[9] Mughal A, O'Rourke ST. Vascular effects of apelin: mechanisms and therapeutic potential. Pharmacol Ther. 2018;190:139‐147. 29807055 10.1016/j.pharmthera.2018.05.013 PMC6165679

[10] Chapman FA, Nyimanu D, Maguire JJ, Davenport AP, Newby DE, Dhaun N. The therapeutic potential of apelin in kidney disease. Nat Rev Nephrol. 2021;17(12):840‐853. 34389827 10.1038/s41581-021-00461-z PMC8361827

[11] Llorens-Cortes C, Moos F. Apelin and vasopressin: two work better than one. J Neuroendocrinol. 2012;24(7):1085‐1086. 22712789 10.1111/j.1365-2826.2012.02316.x

[12] Reaux A, De Mota N, Skultetyova I, et al Physiological role of a novel neuropeptide, apelin, and its receptor in the rat brain. J Neurochem. 2001;77(4):1085‐1096. 11359874 10.1046/j.1471-4159.2001.00320.x

[13] Hurbin A, Boissin-Agasse L, Orcel H, et al The V1aand V1b, but not V2, vasopressin receptor genes are expressed in the supraoptic nucleus of the rat hypothalamus, and the transcripts are essentially colocalized in the vasopressinergic magnocellular neurons. Endocrinology. 1998;139(11):4701‐4707. 9794482 10.1210/endo.139.11.6320

[14] Hus-Citharel A, Bouby N, Frugière A, Bodineau L, Gasc JM, Llorens-Cortes C. Effect of apelin on glomerular hemodynamic function in the rat kidney. Kidney Int. 2008;74(4):486‐494. 18509323 10.1038/ki.2008.199

[15] Nyimanu D, Chapman FA, Gallacher PJ, et al Apelin is expressed throughout the human kidney, is elevated in chronic kidney disease & associates independently with decline in kidney function. Br J Clin Pharmacol. 2022;88(12):5295‐5306. 35748053 10.1111/bcp.15446 PMC9796317

[16] Christ-Crain M, Bichet DG, Fenske WK, et al Diabetes insipidus. Nat Rev Dis Primers. 2019;5(1):54. 31395885 10.1038/s41572-019-0103-2

[17] De Mota N, Reaux-Le Goazigo A, El Messari S, et al Apelin, a potent diuretic neuropeptide counteracting vasopressin actions through inhibition of vasopressin neuron activity and vasopressin release. Proc Natl Acad Sci U S A. 2004;101(28):10464‐10469. 15231996 10.1073/pnas.0403518101 PMC478592

[18] Girault-Sotias P-E, Gerbier R, Flahault A, de Mota N, Llorens-Cortes C. Apelin and vasopressin: the Yin and Yang of water balance. Front Endocrinol (Lausanne). 2021;12:735515. 34880830 10.3389/fendo.2021.735515 PMC8645901

[19] Hus-Citharel A, Bodineau L, Frugière A, Joubert F, Bouby N, Llorens-Cortes C. Apelin counteracts vasopressin-induced water reabsorption via cross talk between apelin and vasopressin receptor signaling pathways in the rat collecting duct. Endocrinology. 2014;155(11):4483‐4493. 25157454 10.1210/en.2014-1257

[20] Christ-Crain M, Fenske W. Copeptin in the diagnosis of vasopressin-dependent disorders of fluid homeostasis. Nat Rev Endocrinol. 2016;12(3):168‐176. 26794439 10.1038/nrendo.2015.224

[21] Blanchard A, Steichen O, De Mota N, et al An abnormal apelin/vasopressin balance may contribute to water retention in patients with the syndrome of inappropriate antidiuretic hormone (SIADH) and heart failure. J Clin Endocrinol Metab. 2013;98(5):2084‐2089. 23515451 10.1210/jc.2012-3794

[22] Flahault A, Girault-Sotias PE, Keck M, et al A metabolically stable apelin-17 analog decreases AVP-induced antidiuresis and improves hyponatremia. Nat Commun. 2021;12(1):305. 33436646 10.1038/s41467-020-20560-y PMC7804859

[23] Refardt J, Imber C, Sailer CO, et al A randomized trial of empagliflozin to increase plasma sodium levels in patients with the syndrome of inappropriate antidiuresis. J Am Soc Nephrol. 2020;31(3):615‐624. 32019783 10.1681/ASN.2019090944 PMC7062212

[24] Refardt J, Imber C, Nobbenhuis R, et al Treatment effect of the SGLT2 inhibitor empagliflozin on chronic syndrome of inappropriate antidiuresis: results of a randomized, double-blind, placebo-controlled, crossover trial. J Am Soc Nephrol. 2023;34(2):322‐332. 36396331 10.1681/ASN.2022050623 PMC10103093

[25] Berezin AA, Fushtey IM, Berezin AE. The effect of SGLT2 inhibitor dapagliflozin on serum levels of apelin in T2DM patients with heart failure. Biomedicines. 2022;10(7):1751. 35885056 10.3390/biomedicines10071751 PMC9313111

[26] Pinheiro J, Bates D, R Core Team. 2022. _nlme: Linear and Nonlinear Mixed Effects Models_. R package version 3.1-157. Accessed November 26, 2023. https://CRAN.R-project.org/package=nlme

[27] Hlavac M. stargazer: Well-Formatted Regression and Summary Statistics Tables. R package version 5.2.1. Accessed November 26, 2023. https://CRAN.R-project.org/package=stargazer

[28] R Core Team . 2022. R: A Language and Environment for Statistical Computing. R Foundation for Statistical Computing. URL https://www.R-project.org/

[29] Monnerat S, Drivakos N. Supplemetary material- Apelin and Copeptin Levels in Patients With Chronic SIAD Treated With Empagliflozin. 2024. Doi: 10.5281/zenodo.11489469 PMC11170659 38872994

[30] Azizi M, Iturrioz X, Blanchard A, et al Reciprocal regulation of plasma apelin and vasopressin by osmotic stimuli. J Am Soc Nephrol. 2008;19(5):1015‐1024. 18272843 10.1681/ASN.2007070816 PMC2386722

[31] Hu PF, Tang JL, Chen WP, Bao JP, Wu LD. Increased apelin serum levels and expression in human chondrocytes in osteoarthritic patients. Int Orthop. 2011;35(9):1421‐1426. 20652246 10.1007/s00264-010-1100-y PMC3167451

[32] Cuesta M, Thompson CJ. The syndrome of inappropriate antidiuresis (SIAD). Best Pract Res Clin Endocrinol Metab. 2016;30(2):175‐187. 27156757 10.1016/j.beem.2016.02.009

[33] Kim S, Jo CH, Kim G-H. The role of vasopressin V2 receptor in drug-induced hyponatremia. Front Physiol. 2021;12:797039‐797039. 34955900 10.3389/fphys.2021.797039 PMC8703040

[34] Refardt J, Atila C, Chifu I, et al Arginine or hypertonic saline-stimulated copeptin to diagnose AVP deficiency. N Engl J Med. 2023;389(20):1877‐1887. 37966286 10.1056/NEJMoa2306263

[35] Fenske W, Refardt J, Christ-Crain M. Copeptin in the diagnosis of diabetes insipidus. N Engl J Med. 2018;379(18):1785‐1786. 30380393 10.1056/NEJMc1811694

[36] Nigro N, Winzeler B, Suter-Widmer I, et al Evaluation of copeptin and commonly used laboratory parameters for the differential diagnosis of profound hyponatraemia in hospitalized patients: ‘The Co-MED study’. Clin Endocrinol (Oxf). 2017;86(3):456‐462. 27658031 10.1111/cen.13243

[37] Fenske WK, Christ-Crain M, Horning A, et al A copeptin-based classification of the osmoregulatory defects in the syndrome of inappropriate antidiuresis. J Am Soc Nephrol. 2014;25(10):2376‐2383. 24722436 10.1681/ASN.2013080895 PMC4178436

[38] Katan M, Christ-Crain M. The stress hormone copeptin: a new prognostic biomarker in acute illness. Swiss Med Wkly. 2010;140:w13101. 20872295 10.4414/smw.2010.13101

[39] Nobbenhuis R, Refardt J, Vogt D, Sailer CO, Winzeler B, Christ-Crain M. Can treatment response to SGLT2-inhibitors in syndrome of inappropriate antidiuresis be predicted by copeptin, natriuretic peptides and inflammatory markers? Biomarkers. 2021;26(7):647‐655. 34412521 10.1080/1354750X.2021.1970808

[40] Lytvyn Y, Bjornstad P, Katz A, et al SGLT2 inhibition increases serum copeptin in young adults with type 1 diabetes. Diabetes Metab. 2020;46(3):203‐209. 31816431 10.1016/j.diabet.2019.11.006 PMC7253338

[41] Berton AM, Parasiliti-Caprino M, Prencipe N, et al Copeptin adaptive response to SGLT2 inhibitors in patients with type 2 diabetes mellitus: the GliRACo study. Front Neurosci. 2023;17:1098404. 37021137 10.3389/fnins.2023.1098404 PMC10067557

[42] Chapman FA, Maguire JJ, Newby DE, Davenport AP, Dhaun N. Targeting the apelin system for the treatment of cardiovascular diseases. Cardiovasc Res. 2023;119(17):2683‐2696. 37956047 10.1093/cvr/cvad171 PMC10757586

[43] Vart P, Vaduganathan M, Jongs N, et al Estimated lifetime benefit of combined RAAS and SGLT2 inhibitor therapy in patients with albuminuric CKD without diabetes. Clin J Am Soc Nephrol. 2022;17(12):1754‐1762. 36414316 10.2215/CJN.08900722 PMC9718016

[44] Bonora BM, Avogaro A, Fadini GP. Extraglycemic effects of SGLT2 inhibitors: a review of the evidence. Diabetes Metab Syndr Obes. 2020;13:161‐174. 32021362 10.2147/DMSO.S233538 PMC6982447

[45] Verma S, McMurray JJV. SGLT2 inhibitors and mechanisms of cardiovascular benefit: a state-of-the-art review. Diabetologia. 2018;61(10):2108‐2117. 30132036 10.1007/s00125-018-4670-7

[46] Sourij H, Aziz F, Mangge H, von Lewinski D. SGLT2 inhibition could potentially impact inflammation in acute myocardial infarction. Eur Heart J. 2023; 44(38):3931‐3931. 37350395 10.1093/eurheartj/ehad404

